# Assessment of the bioactive compounds in gamma irradiated stevia (*Stevia rebaudiana Bertoni*) leaves

**DOI:** 10.1186/s12896-025-01008-x

**Published:** 2025-07-16

**Authors:** Noha Eid Eliwa, Mohamed Farouk Ahmed

**Affiliations:** Natural Products Department, National Centre for Radiation Research and Technology, Egyptian Atomic Energy Authority, Cairo, Egypt

**Keywords:** Herbs, Ionizing radiation, HPLC chromatogram, Sugar, Phenols, Protein

## Abstract

Stevia is a potential alternative sweetener for individuals with diabetes. Gamma radiation is one technique that can alter a plant’s physiological traits or phytochemical makeup without producing any dangerous byproducts or chemical initiators. Therefore, the aim of the current study was to determine the effect of gamma radiation (0, 3, 5, 7, and 10 kGy) on the bioactive compounds of dry stevia leaves. In comparison to non-irradiated samples, it is clear that all gamma radiation doses raised the percentages of carbohydrates, total steviosides, total sugar, reducing sugar, crude protein, and nitrogen, while decreasing the percentages of fat, ash, and fiber. The irradiation of stevia leaves at a dose of 7 kGy resulted in the most significant increase in carbohydrates by 57.7%, total steviosides by 32.8%, total sugars by 38%, reduced sugars by 66.8%, and crude protein by 21.9% when compared to non-irradiated samples. In contrast, the percentages of fat, ash, and fiber decreased by 23.2%, 10.8%, and 11.9%, respectively. According to the HPLC profile chromatogram, stevia leaves exposed to 3, 5, and 7 kGy had higher concentrations of all identified phenolic compounds than non-irradiated leaves; 5 kGy was outperformed by 3 and 7 kGy, while 10 kGy resulted in a decrease in these compounds. While apigenin and ellagic acid only disappeared from leaves exposed to 10 kGy, kaempferol was seen to disappear from all irradiated leaves. Furthermore, cinnamic acid was detected at radiation doses of 5, 7, and 10 kGy (0.50, 0.90, and 0.14 µg.ml^− 1^, respectively), whereas it was absent at the non-irradiated and 3 kGy radiation doses. The Fourier Transform Infrared (FTIR) spectra of the irradiated and non-irradiated stevia samples displayed a comparable band profile. In conclusion, gamma irradiation of dried stevia leaves increased the levels of carbohydrates, steviosides, sugars, crude protein, and phenolic compounds, while reducing the levels of fat, ash, and fiber, with no observable differences in the FTIR spectra between the irradiated and non-irradiated samples. The optimal radiation dose was 7 kGy, which resulted in the most significant enhancement in biologically active compounds, along with the emergence of cinnamic acid.

## Introduction

Stevia (*Stevia rebaudiana Bertoni*), a bushy shrub of the Asteraceae family, originates from Brazil and Paraguay and is cultivated worldwide [1]. It contains a wealth of nutrients, including dietary fibers, proteins, fats, ash, and carbohydrates [2]. This plant is also rich in a number of minerals, including K, Ca, Na, Mg, Cu, Mn, Fe, and Zn, and it contains more essential and non-essential amino acids than the FAO and WHO recommend [3]. Stevia, sometimes referred to as honey leaf, is regarded as a good sugar alternative. Steviol glycosides, which are extracted from the stevia plant, are thought to be a very beneficial component in preventing diabetes and obesity because they enhance insulin sensitivity and secretion [4]. This could be explained by the fact that the sweetener’s compounds are difficult for the human digestive system to digest and cannot be broken down chemically. As a result, it is a safe sweetener for people, especially diabetics [5]. Steviosides are sugar-like substances that are 200–300 times more potent than sucrose, low in calories, non-toxic, and non-mutagenic, and that have FDA approval [6]. The plant Stevia rebaudiana provides secondary metabolites with diterpene glycosides, which are also known as steviol glycosides. The primary steviol glycoside found in *Stevia rebaudiana* leaves is stevioside, which is followed by glucoside A, Rebaudiana A, and Rebaudiana C sweetener [7]. These substances are not only responsible for the sweet flavor of Stevia rebaudiana leaves but also have anti-inflammatory, anti-diabetic, antihypertensive, anticancer, anti-atherosclerotic, and diuretic properties, making them potentially useful for treating a number of illnesses [4, 7]. Apart from glycosides, phenols, and flavonoids found in stevia leaf extracts give the plant extracts their antioxidant qualities. The phenolic compounds found in stevia leaves that have the highest antioxidant efficacy are diosmin, rutin, ferulic acid, caffeic acid, ellagic acid, chlorogenic acid, isochlorogenic acid, and other hydroxycinnamic acids. Additionally, it contains oligosaccharides, free sugars, amino acids, lipids, alkaloids, chlorophylls, xanthophyll, and trace elements [8–10].

When it comes to changing physiological characteristics or phytochemical composition, gamma radiation can be helpful [11, 12]. Since gamma irradiation produces no hazardous byproducts or chemical initiators, it is gaining a lot of attention for its technical utility and effectiveness in producing degraded products. It is an effective physical method for changing polysaccharides via degradation, grafting, and cross-linking [13]. Moreover, high-dose radiation can disrupt lignocellulose’s structure and boost enzymatic hydrolysis’s effectiveness, which will accelerate the dissolution of water-soluble carbohydrates [14]. Earlier research has demonstrated that the effects of radiation on the physicochemical characteristics of various food types are rather intricate, as the dosage and kind of irradiation, along with the type of food, alter this impact [15]. In this context, the gamma irradiation (1, 5, and 8 kGy) applied to Sacha Inchi (*Plukenetia volubilis* L.) seeds did not lead to any significant changes in the physical properties and chemical composition of Sacha Inchi oils [16]. Conversely, the irradiation of *Ginkgo biloba* L. at 1 and 10 kGy enhanced the extractability of phenolic compounds in both methanol/water and infusion preparations [17]. Consequently, studies examining the impact of radiation must be performed for every product of interest [15].

Given that the application of gamma rays to improve the chemical properties of stevia leaves remains a subject of research, the objective of this study is to assess the impact of gamma radiation (3, 5, 7, and 10 kGy) on different nutritional value metrics of dried stevia leaves, along with the alterations in phenolic compounds. It is anticipated that the findings will be beneficial for the creation of innovative food supplements.

## Materials and methods

### Plant material

In 2021, dried stevia (*Stevia rebaudiana Bertoni*) leaves were bought online from Green Stevia (www.greengreenstevia.com). The leaves of stevia were divided into five uniform groups, each weighing 90 g, which were further partitioned into three replicates of 30 g each arranged in 3 samples of 10 g each, subsequently placed in polyethylene bags prior to exposure to radiation.

### Chemicals

Folin-Ciocalteu reagent and chemical HPLC-grade standards (purity > 95%) were acquired from Sigma-Aldrich (St. Louis, MO, USA). Other reagents utilized in the experiments were of analytical grade.

### Irradiation process

The dried stevia leaves were subjected to gamma radiation at varying doses of 3, 5, 7, and 10 kGy [18, 19] utilizing a Gamma Cell (^60^Co) with a dose rate of 0.84 kGy.h^− 1^. This irradiation procedure was conducted at the National Center for Radiation Research and Technology, Egyptian Atomic Energy Authority, located in Cairo, Egypt. The samples that were not exposed to irradiation were preserved as control groups at ambient temperature (~ 27 °C, the same temperature at which irradiation was performed). Both the non-irradiated and irradiated samples underwent analysis within a one-week timeframe (room temperature ~ 27 °C, humidity ~ 70%).

### Proximate composition

The percentages of total carbohydrates, reduced sugars, and total sugars in the dried stevia leaves were determined in accordance with [20]. The results were made as mg.100 g^− 1^ of stevia leaf dry weight. The percentage of non-reducing sugars was calculated by subtracting the total sugars from the reducing sugars.

The percentage of total stevioside in dried leaves was calculated using the modified Anthrone-sulfuric acid method [21]. To make the anthrone reagent, 0.2 g of anthrone was dissolved in 100 mL of H_2_SO_4_. When needed, this reagent was made fresh. Two milliliters of the extract sample were then combined with six milliliters of anthrone solution and shaken vigorously. To stop the tube from losing water through evaporation, it was submerged in an ice bath. The tube was cooled and then brought to a boil for ten minutes before being cooled once more under running water. The test tubes were incubated at room temperature (27 °C) for 30 min. Reagents and distilled water were used to maintain Blank. At 630 nm, the variations in the green solution’s absorbance were measured. By comparing the absorbance of the sample with that of the stevioside standard, the total stevioside percentage was calculated.

The percentages of fat, ash, and fiber in the prepared samples were determined using the method outlined in [22]. To determine the N percentage, the leaves were oven-dried at 70 °C until the samples’ weight stayed constant, and then they were ground into a powder mixture. Following the digestion of 0.2 g of the dried samples with concentrated sulfuric acid, the nitrogen content was determined using modified micro Kjeldahl techniques [23]. The nitrogen content was multiplied by 6.25 to determine the percentage of crude protein [22].

### Phenolic compound determination by high-performance liquid chromatography (HPLC)

Stevia leaves’ profile of phenolic compound contents (µg.ml^− 1^) was ascertained using an Agilent 1260 series HPLC chromatogram. For the separation, an Eclipse C18 column (4.6 mm x 250 mm i.d., 5 μm) was used. Water (A) and 0.05% trifluoroacetic acid in acetonitrile (B) at a flow rate of 0.9 ml/min made up the mobile phase. A linear gradient was used to program the mobile phase in the following order: 0 min (82% A); 0–5 min (80% A); 5–8 min (60% A); 8–12 min (60% A); 12–15 min (82% A); 15–16 min (82% A); and 16–20 (82% A). Monitoring of the multi-wavelength detector took place at 280 nm. For each sample solution, the injection volume was 5 µL. The temperature of the column was kept at 40 °C. Samples and standard solutions underwent filtration through 0.45 μm hydrophilic PTFE membrane filters before being injected. The identification of the compounds in the chromatograms was achieved by comparing their retention times with those of reference standards. Each phenolic compound was determined using the relevant calibration curve. Extract samples were analyzed by injecting them into HPLC three times.

### Fourier transform infrared (FTIR) spectroscopy

The characteristic functional groups in stevia samples were identified using FTIR spectroscopy between 4000 and 400 cm^− 1^ at a resolution of 4 cm^− 1^ using the Bruker Vertex 70 FT-IR spectrometer, which is linked to a HYPERION™ series microscope (Bruker Optik GmbH, Ettlingen, Germany). A dry constant weighted sample was mixed with 3 mg of KBr and pressed to form a transparent disk [24].

### Statistical analysis

A randomized complete block design was used for this experiment (5 treatments x 3 replicates x 3 samples/replicate). One-way analysis of variance (ANOVA) was performed on the data. Duncan’s multiple range tests (*P* < 0.05) were used to compare the means using the newly established L.S.D. values at the 5% level [25]. The statistical analysis was conducted using the M-STAT computer program [26].

## Results and discussion

### Proximate composition of non-irradiated and irradiated stevia leaves

The effects of gamma irradiation on the different biochemical components of stevia leaves were explained in Table 1. It is evident that different gamma radiation doses had a comparable impact on the percentages of carbohydrates and total steviosides. The different gamma doses significantly increased the percentages of carbohydrates and total steviosides as the radiation dose increased in comparison to non-irradiated leaves, with no detectable differences between 7 and 10 kGy. Carbohydrate and total stevioside percentages increased by 57.7% and 32.8 for 7 kGy and 49.4% and 31.3% for 10 kGy when compared to non-irradiated leaves.

All applied gamma radiation doses significantly increased the percentages of total sugar and reducing sugar in the leaves when compared to non-irradiated leaves. The dose of 7 kGy produced the highest increase in total sugar, followed by 5 kGy, 10 kGy, and then 3 kGy. On the other hand, the dose of 7 kGy produced the highest increase in reducing sugar, followed by 10 kGy, 5 kGy, and then 3 kGy, while non-irradiated leaves produced the lowest value. The dose of 5 kGy produced the highest value for non-reducing sugar, followed by 10 kGy, 0 kGy, 3 kGy, and 7 kGy, which produced the lowest value. The greatest increase in total sugar was 38% at 7 kGy and 29.5% at 5 kGy, whereas the greatest increase in reducing sugar was 66.8% at 7 kGy and 38.6% at 10 kGy in comparison to stevia leaves that were not exposed to radiation. The results demonstrated that gamma radiation increases mono and disaccharides by degrading polysaccharides.

In contrast to sugar, stevia leaves exposed to gamma radiation had lower percentages of fat, ash, and fiber than leaves that were not exposed to radiation. With increasing radiation dose, the decrease increases exponentially; the lowest values were obtained with 10 kGy, while the highest values were obtained with non-irradiated leaves. When compared to non-irradiated leaves, the percentages of fat, ash, and fiber reduction were 27.3%, 25%, and 24.6% at 10 kGy, and 23.2%, 10.8%, and 11.9% at 7 kGy, respectively.

**Table 1:** The effect of different doses of gamma radiation on some bioactive compounds of dry stevia leaves extract

Radiation dose	0 kGy	3 kGy	5 kGy	7 kGy	10 kGy
Carbohydrates%	28.33 ^d^	34.33 ^c^	37.33 ^b^	44.67 ^a^	42.33 ^a^
Total steviosides%	6.00 ^d^	6.50 ^c^	7.03 ^b^	7.97 ^a^	7.88 ^a^
Total sugar%	8.57 ^e^	8.95 ^d^	11.10 ^b^	11.83 ^a^	10.84 ^c^
Reducing sugar%	5.33 ^e^	5.75 ^d^	5.98 ^c^	8.89 ^a^	7.39 ^b^
Non-reducing sugar%	3.24 ^c^	3.20 ^d^	5.12 ^a^	2.94 ^e^	3.45 ^b^
Fat%	5.42 ^a^	5.14 ^b^	4.82 ^c^	4.16 ^d^	3.94 ^e^
Ash %	7.52 ^a^	7.42 ^b^	6.88 ^c^	6.71 ^d^	5.64 ^e^
Fiber%	8.39 ^a^	8.18 ^b^	7.77 ^c^	7.39 ^d^	6.33 ^e^
N%	1.14 ^e^	1.25 ^d^	1.32 ^b^	1.38 ^a^	1.27 ^c^
Crude protein%	7.13 ^e^	7.79 ^d^	8.26 ^b^	8.64 ^a^	7.92 ^c^

Means with different letters are significantly different at *p* < 0.05

The data showed that the percentages of crude protein and N all responded to gamma irradiation similarly to how total sugar did. In comparison to leaves that were not exposed to gamma irradiation, the percentages of N and crude protein increased significantly in response to varying gamma irradiation doses. Up to 7 kGy, where the highest value was obtained, the percentages of N and crude protein increased significantly. After that, the values dropped to a level below that of 5 kGy. Stevia leaves exposed to 7 kGy had the largest increase in N and crude protein (21.1%), followed by those exposed to 5 kGy (15.8%).

According to our data, gamma irradiation of stevia leaves resulted in a significant increase in the percentages of crude protein, sugars, steviosides, carbohydrates, and nitrogen while decreasing the percentages of fat, ash, and fibers. The most effective dose was 7 kGy. Gamma irradiation of stevia leaves at 5 and 10 krad markedly raised the content of steviol glycosides [27]. Also, irradiating stevia callus culture with a dose of 20 Gy enhanced the antioxidant capacity; while a dose of 15 Gy was found to increase the content of stevia callus of stevioside and total flavonoids [28]. Additionally, stevia leaves exposed to 0.5, 1, and 2 kGy of gamma irradiation had significantly lower fat contents than commercial leaves, but their ash content remained unaffected [29]. On the other hand, Colocasia leaves exposed to 0.5–2.5 kGy gamma radiation showed a dose-dependent decrease in the proportion of ash and fibers, but the fat content remained unchanged [30]. Radiation-induced fat oxidation [31] and decreased activity of the enzymes involved in the de novo synthesis of fatty acids [32] may be the cause of the lower fat content. In terms of sugar content, gamma irradiation improved the amount of free sugars in dried rose hip fruits [33] and soluble sugars in sugarcane [34]. They also mentioned that there was no discernible impact from the higher dose (25 kGy), but the increment was noticeable at a lower dose (10 kGy). They attributed this change to the modulation of the activity of enzymes involved in carbohydrates, which resulted in the conversion of some of the cellulose and starch into glucose and sucrose. It was recorded that, in comparison to non-irradiated samples, irradiation doses ranging from 0.5 to 2 kGy increased the fat and fiber content of mango peels while maintaining the same protein content [35]. Also, irradiation had a dose-dependent effect on the amount of ash; low doses had no effect, while 1.5 kGy and 2 kGy doses increased and decreased the content, respectively. Every dosage raised the amount of sugar, but only the 2 kGy increase was noticeable. In addition, the application of gamma irradiation to raw cocoa beans at doses of 1, 3, 5, and 7 kGy did not significantly affect the protein and moisture content; however, the fat and ash content showed a significant decline at higher doses [18]. One possible explanation for the discrepancy with our data is the use of different species and lower irradiation doses. Radiation treatment improved biological characteristics, raised levels of beneficial phytochemicals, and had a positive effect on biomolecules such as proteins, lipids, carbohydrates, and other phytochemicals by causing structural and chemical changes [36, 37]. The chemical composition of starch varies from species to species; it generally has a similar chemical composition. Because gamma modification causes less of a change in chemical composition, the food industry can safely use this technique to improve the quality of starch and foods that contain it without creating hazardous by-products [38]. The plant stevia has a lot of health benefits. It has several metabolites with antioxidant qualities in addition to steviol glycosides, which are used as natural sweeteners that don’t contain calories [39]. One processing method that attempts to increase food security is food irradiation. The administered dose, the specificity of the product, and the sensitivity of each phytochemical will all affect how radiation treatment affects antioxidant and phytochemical levels [40].

### Phenolic compounds of non-irradiated and irradiated stevia leaves

Table 2 shows a list of the phenolic compounds in the stevia leaves affected by gamma radiation through HPLC analysis. In stevia leaves extracts 18 phenolic compounds were identified. It is clear all phenolic compounds increased with 3 kGy radiation above all treatments studied, with the exception of cinnamic acid and kaempferol which disappeared. It is important to note that kaempferol compounds disappeared with all irradiated leaves. Gamma radiation at doses of 5, 7 and 10 kGy show the production of cinnamic acid compounds (0.50, 0.90 and 0.14 µg/ml, respectively), as compared to control and 3 kGy irradiated leaves i.e. (0 µg/ml) for both treatments. In addition, apigenin compounds disappeared only with 10 kGy irradiated leaves. In general, radiation dose of 7 KGy caused increase in phenolic compounds in leaves than 5 and 10 kGy radiation doses.

Gamma irradiation of dried leaves of *Khaya senegalensis*, *Euodia malayana*, and *Gnetum gnemon* significantly increased the proportion of the total content of phenolic acid. Additionally, as the gamma radiation dose increased (9–13 kGy), the gallic acid concentration ascended noticeably [41]. Methanolic extracts of irradiated thyme (5, 10, and 15 kGy) had higher levels of total phenols and flavonoids than non-irradiated thyme. Dose of 10 kGy produced the highest content, followed by 5 kGy and 15 kGy [42]. Additionally, *Satureja mutica*’s highest phenol content and antioxidant capacity were achieved after the plants were exposed to 2.5 and 7.5 kGy gamma rays [19]. It was found that in irradiated peppermint samples, the concentration of all phenolic compounds increased significantly. Compared to the non-irradiated sample, samples exposed to 5 and 10 kGy radiation had the highest concentrations of total phenolic acids, total flavonoids, and total phenolic compounds. For lemon verbena, samples exposed to 1 kGy had higher levels of total phenolic acids and total phenolic compounds, whereas samples exposed to 10 kGy had higher levels of total flavonoids [43].

**Table 2:** The effect of different doses of gamma radiation on the profile of phenolic compounds contents (µg.ml^− 1^) of dry stevia leaves extract

	0 kGy	3 kGy	5 kGy	7 kGy	10 kGy
Gallic acid	14.85	20.51	16.24	19.63	12.61
Chlorogenic acid	528.41	810.77	673.71	807.06	539.25
Methyl gallate	0.27	0.92	0.25	1.05	0.20
Coffeic acid	3.61	6.39	4.20	6.14	3.46
Syringic acid	0.70	1.15	0.79	0.98	0.64
Pyro catechol	3.74	9.79	4.41	5.51	2.68
Rutin	32.09	39.92	33.57	38.62	24.36
Ellagic acid	0.96	3.86	1.39	6.45	0.00
Coumaric acid	0.78	1.13	1.13	0.95	2.09
Vanillin	1.62	1.88	2.00	2.88	1.42
Ferulic acid	16.28	21.57	18.26	21.84	13.57
Naringenin	479.22	706.46	606.35	714.00	453.41
Daidzein	4.03	7.87	4.86	6.42	3.13
Quercetin	2.17	3.12	3.22	3.68	2.66
Cinnamic acid	0.00	0.00	0.50	0.90	0.14
Apigenin	12.04	15.54	6.19	11.96	0.00
Kaempferol	1.07	0.00	0.00	0.00	0.00
Hesperidin	0.43	0.77	0.33	2.72	0.19

According to another study, depending on the applied dose (0, 1, and 10 kGy) and the specific compounds, gamma irradiation of methanolic extracts of lemon verbena increases the concentration of some phenolic compounds and decreases that of others [44]. The increased total phenolic content following gamma irradiation was also documented in peanut skin [45], *Prunus amygdalus* skin extracts [46], and water extracts of *Rosmarinus officinalis L.* [47]. Buckwheat flour and grain contents that were exposed to gamma radiation (2, 4, 6, 8, and 10 kGy) demonstrated greater total phenolic and total flavonoid when compared to samples that were not irradiated, in addition to increased rutin levels, with a direct correlation between the radiation dose and the content [48]. The natural phenolic compounds known as caffeoylquinic acid derivatives were markedly elevated in dried *Pluchea indica* leaf powder subjected to irradiation of 5–10 kGy [49]. Gamma irradiation has shown a significant impact on the total phenolic content, with reports indicating that higher doses of irradiation notably enhance the total phenolic content in irradiated stevia leaves. This enhancement may be attributed to the effects of irradiation, which alters chemical bonds and breaks down larger compounds into smaller fragments, thereby releasing them from their matrix structures [50, 51]. Similarly, other research has linked this alteration to the stimulation of phenylalanine ammonia-lyase biosynthesis, which promotes the synthesis of phenolic compounds. Moreover, radiation-induced depolymerization of polysaccharides aids in the release of phenols [52], as well as the depolymerization and dissolution of the cell wall, which enhances the extractability of various compounds [53].

### Functional groups of non-irradiated and irradiated stevia leaves

**Fig. 1:**
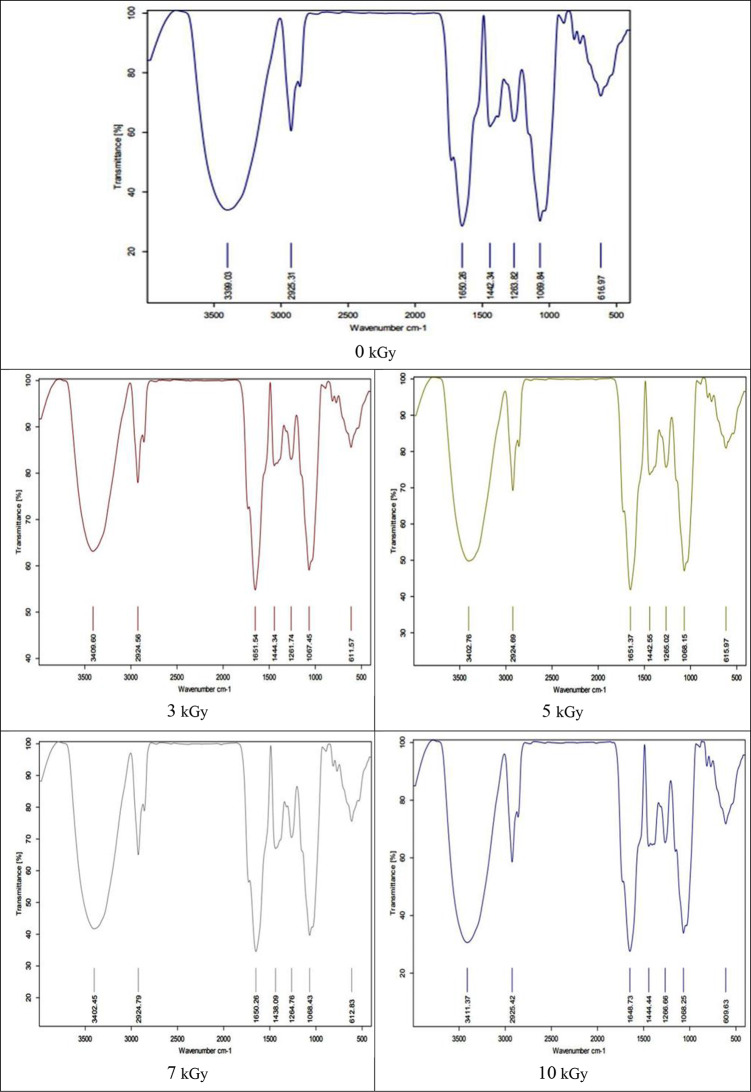
FTIR spectra of dry stevia leave powder affected by different doses of gamma irradiation

The FTIR spectra of stevia leaf powder were recorded in order to characterize various functional groups present, and gather sufficient information about the dried powder of stevia. The IR spectra for raw non-irradiated stevia powder give different bands indicating the particular functional groups at distinct IR wavelengths. In general, FTIR profiles from the different stevia leaves (Table 3; Fig. 1) indicated the presence of the peak near 3400 cm^− 1^ (3400–3200 cm^− 1^), mainly due to the O-H bending vibrations, which is associated with the presence of the polyphenol. The stevia FTIR spectrum also showed asymmetric and symmetric stretching vibrations of -CH appearing at 2940 and 2925 cm^− 1^ indicating the presence of carbohydrates and polysaccharides, which is the characteristic band. The ketones and aromatic bonds were observed in the band 1615–1580 cm^− 1^. The band around 1400 cm^− 1^ is also assigned to the stretching vibration of the C-O bond, indicating the presence of phenolic. Bands at 1200 cm-1 IR spectrum are attributed to RCOOR`stretching of the ester groups. At 1065–1000 cm^− 1^, a broad band indicated to ether group, C-O of steviol and glycoside were the characteristic absorption band of the glycosidic bond. Alkene C = C and C- Br were observed at 600 cm^− 1^.

**Table 3:** The effect of different doses of gamma radiation on the functional groups in stevia leaves powder (FTIR spectroscopy)

Wave length	Function group	0 kGy	3 kGy	5 kGy	7 kGy	10 kGy
3400 − 3200	-OH stretching N-H (poly phenol, Alcohols and secondary amides)	3399.03	3409.60	3402.76	3402.45	3411.37
3000 − 1700	C-H/CH_2_/CH_3_ (Alkane, Carbohydrate and polysaccharides	2925.31	2924.56	2924.69	2924.79	2925.42
1650 − 1580	C-H/C = C-C, C = O (Ketones, aromatic bonds)	1650.26	1651.54	1651.37	1650.26	1648.73
1400 − 1300	C-O phenolic	1442.34	14444.34	1442.55	14308.09	1444.44
1200	Esters (RCOOR`)	1263.82	1261.71	1265.02	1264.76	1266.66
1065 − 1000	C-O-C stretching (cm − 1) ether groups corresponded to C O derived from steviol and glycoside were characteristic absorption band of the glycosidic bond	1069.84	1067.45	1068.15	1068.43	1068.25
600	C = C Alkenes / C-Br	616.97	611.57	615.97	612.83	609.63

Alterations and changes in biochemical profiles are commonly characterized by FTIR spectroscopy. FTIR is also useful for identifying the functional groups; the minor and major changes present in primary and secondary metabolites as well as biomolecules such as DNA, RNA, protein, carbohydrate, and lipids. Also, it generates the spectrum for the biochemical and metabolites of the samples [54, 55]. Our results regarding the FTIR profile of non-irradiated stevia leaves were consistent with experiments on stevia grown in Pakistan [56]. They discovered a wide band at 3301.05 cm^− 1^ that indicates the presence of secondary amide groups that reflected protein availability as well as alcohols with OH groups stretching. However, the peaks at 2848.45 cm-1 and 2920.71 cm^− 1^, respectively, show sp2 and sp3 hybridization of carbon. This suggests that compounds with alkane functional groups and configurations are present. The ketone C = O stretching group components, which are linked to flavor along with various aldehyde groups, are indicated by the 1604.74 cm^− 1^ band in the IR spectra of powdered stevia leaves. The C = C stretching at 1509.66 cm^− 1^ has revealed the presence of alkenes and primary amines, which are crucial constituents of all steviosides. Bending of -OH-groups, a crucial component of various chemical groups, including glucose attached to the steviol, which is thought to be the fundamental building block of all steviosides, has been observed at 1372.74 cm^− 1^. RCOOR ~ stretching of ester groups, alkanes (C-C), and carboxylic groups (ROOH) is responsible for the bands in the infrared spectrum located at 1022 cm^− 1^ and 809.84 cm^− 1^, respectively. The C-H aromatic and heteroaromatic compounds are observed in the region 3000–3100 cm-1. The ring C-C stretching vibration appears in the region 1625–1430 cm^− 1^. The C = 0 stretching using FTIR are assigned at 1659 and 1662 cm^− 1^. The O-H stretching vibrations are located at 3238, 3209 and 3132 cm^− 1^. The O-H stretching of Stevia Rebaudiana shows the unique antioxidant potential [57]. A similar band profile to our results was reported by [29] for stevia leaves. They also mentioned that irradiating stevia leaves up to 2 kGy produced a band profile that was similar to that of non-irradiated leaves.

## Conclusions

Gamma irradiation applied to dried stevia leaves led to an increase in the levels of carbohydrates, steviosides, sugars, and crude protein, while simultaneously decreasing the levels of fat, ash, and fiber when compared to non-irradiated samples. The application of a 7 kGy dose resulted in the most pronounced increase in carbohydrates (57.7%), total steviosides (32.8%), total sugars (38%), reduced sugars (66.8%), and crude protein (21.9%) in comparison to non-irradiated samples. Conversely, the percentages of fat, ash, and fiber experienced reductions of 23.2%, 10.8%, and 11.9%, respectively. HPLC analysis indicated a notable increase in the content of phenolic compounds in the irradiated stevia leaves. The optimal radiation dose identified was 7 kGy, which resulted in the most significant enhancement of biologically active compounds, alongside the appearance of cinnamic acid. The FTIR spectra did not reveal any discernible differences between the irradiated and non-irradiated samples. Consequently, gamma irradiation, especially at 7 kGy, could be employed as a potent approach for the preservation of stevia leaves along with improved nutritional quality.

## Data Availability

The author confirms that all data generated or analyzed during this study are included in this published article.
